# Supplementary material for: Tracing Viral Transmission and Evolution of Bovine Leukemia Virus through Long Read Oxford Nanopore Sequencing of the Proviral Genome
Source: Pathogens. 2021 Sep 14;10(9):1191. doi: 10.3390/pathogens10091191 (PMC8470207; doi:10.3390/pathogens10091191)
Supplement: Supplementary file 1 [file pathogens-10-01191-s001.zip › pathogens-1343062-supplementary.pdf]

pathogens

MDPI

Supplementary Materials:

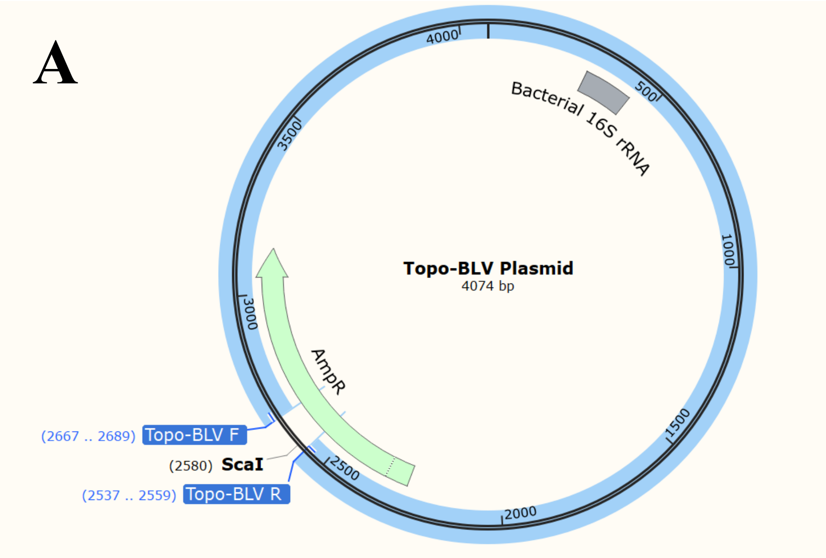

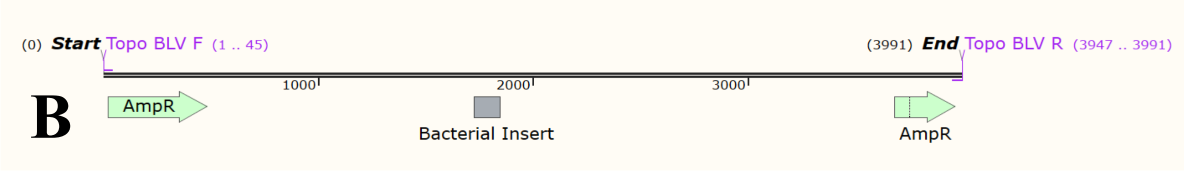

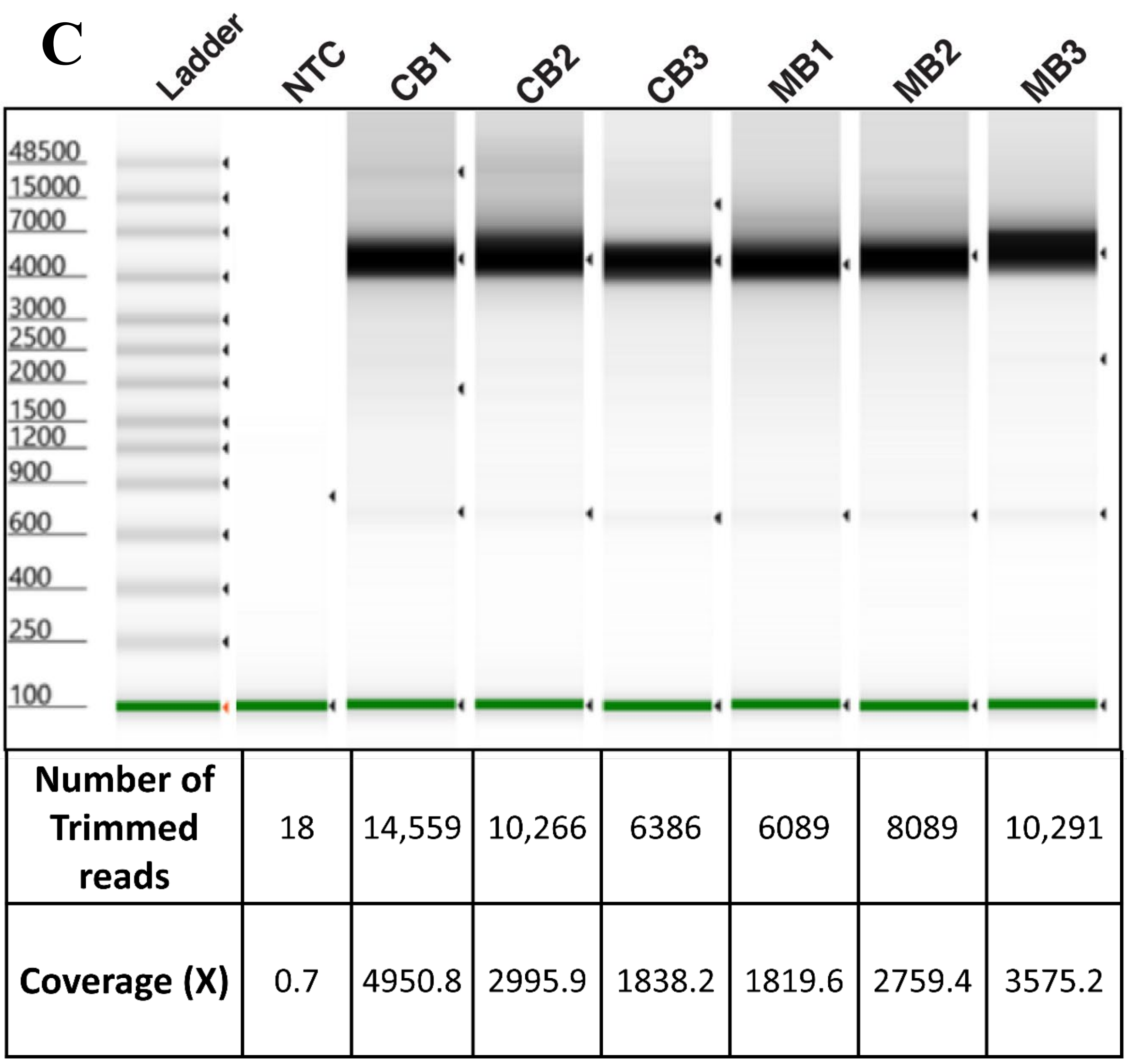

**Number of Trimmed reads**	18	14,559	10,266	6386	6089	8089	10,291
**Coverage (X)**	0.7	4950.8	2995.9	1838.2	1819.6	2759.4	3575.2

Figure S1: (**A**): Map of the TOPO ® vector used with Corynebacterium bovis and Mycoplasma bovis inserts. (**B**): Primary amplicon of Table 1. used as a template for controls. Amplicon contains BLV seed sequence for use as a positive control in the BLV PCR. (**C**): Electrophoretogram showing the amplification, size, and integrity of TOPO ® plasmids encoding the 16s rDNA gene and Thioredoxin gene from two different pathogens, respectively, used for Oxford Nanopore Sequencing. These amplicons were generated using the BLV_CS primers before sequencing. (CB: Corynebacterium bovis; MB- Mycoplasma bovis; NTC: Non-Template Control). NTC: Non-template Control.

```
                      10         20         30         40         50         60         70         80         90         100        110        120
Consensus      CAKSPRYTLD SVNGYPKIYW PPPQGRRRFG ARAMVTYDCE PRCPYVGADR FDCPHWDNAS QADQGSFYVN HQILFLHLKQ CHGIFTLTWE IWGYDPLITF SLHKIPDPPQ PDFPQLNSDW
AP019598       .......... .......... .......... .......... .......... .......... .......... .......... .......... .......... .......... ..........
Dam 1_CS       .......... .......... .......... .......... .......... .......... .......... .......... .......... .......... .......... ..........
Dam 3_CS       .......... .......... .......... .......... .......... .......... .......... .......... .......... .......... .......... ..........
Daughter 3_CS  .......... .......... .......... .......... .......... .......... .......... .......... .......... .......... .......... ..........
Dam 2_CS       .......... .......... .......... .......... .......... .......... .......... .......... .......... .......... .......... ..........
Daughter 2_CS  .......... .......... .......... .......... .......... .......... .......... .......... .......... .......... .......... ..........
Dam 6_CS       .......... .......... .......... .......... .......... .......... .......... .......... .......... .......... .......... ..........
Dam 7_CS       .......... .......... .......... .......... .......... .......... .......... .......... .......... .......... .......... ..........
Daughter 4_CS  .......... .......... .......... .......... .......... .......... .......... .......... .......... .......... .......... ..........
Daughter 8_CS  .......... .......... .......... .......... .......... .......... .......... .......... .......... .......... .......... ..........
Daughter 1_CS  .......... .......... .......... .......... .......... .......... .......... .......... .......... .......... .......... ..........
Daughter 6_CS  .......... .......... .......... .......... .......... .......... .......... .......... .......... .......... .......... ..........
Daughter 7_CS  .......... .......... .......... .......... .......... .......... .......... .......... .......... .......... .......... ..........
Daughter 5_CS  .......... .......... .......... .......... .......... .......... .......... .......... .......... .......... .......... ..........
Dam 5_CS       .......... .......... .......... .......... .......... .......... .......... .......... .......... .......... .......... ..........
Dam 4_CS       .......... .......... .......... .......... .......... .......... .......... .......... .......... .......... .......... ..........

                      130        140        150        160        170        180        190
Consensus      VPSVRSWALL LNQTARAFPD CAICWEPSPP WAPEILVYNK TISSSGPGLA LPDAQIFWVN TSSFNTTQGW HHPSQRLL
AP019598       .......... .......... .......... .......... .......... .......... .......... ........
Dam 1_CS       .......... .......... .......... .......... .......... .......... .......... ........
Dam 3_CS       .......... .......... .......... .......... .......... .......... .......... ........
Daughter 3_CS  .......... .......... .......... .......... .......... .......... .......... ........
Dam 2_CS       .......... .......... .......... .......... .......... .......... .......... ........
Daughter 2_CS  .......... .......... .......... .......... .......... .......... .......... ........
Dam 6_CS       .......... .......... .......... .......... .......... .......... .......... ........
Dam 7_CS       .......... .......... .......... .......... .......... .......... .......... ........
Daughter 4_CS  .......... .......... .......... .......... .......... .......... .......... ........
Daughter 8_CS  .......... .......... .......... .......... .......... .......... .......... ........
Daughter 1_CS  .......... .......... .......... .......... .......... .......... .......... ........
Daughter 6_CS  .......... .......... .......... .......... .......... .......... .......... ........
Daughter 7_CS  .......... .......... .......... .......... .......... .......... .......... ........
Daughter 5_CS  .......... .......... .......... .......... .......... .......... .......... ........
Dam 5_CS       .......... .......... .......... .......... .......... .......... .......... ........
Dam 4_CS       .......... .......... .......... .......... .......... .......... .......... ........
```

Figure S2. Amino acid alignment of the ENV protein showing 100% identity between the animals studied. Only sequences derived from BLV amplicons generated by CentralStar Laboratories were used for this analysis. AP019598 is the Genbank accession number of the reference genome used for comparison.
